# Clinical decision-making: physicians' preferences and experiences

**DOI:** 10.1186/1471-2296-8-10

**Published:** 2007-03-15

**Authors:** Elizabeth Murray, Lance Pollack, Martha White, Bernard Lo

**Affiliations:** 1 Department of Primary Care and Population Sciences, Royal Free and University College Medical School at University College London, Archway Campus, Highgate Hill, London N19 5LW, UK; 2 Health Survey Research Unit, Center for AIDS Prevention Studies, University of California, San Francisco, 74 New Montgomery, Suite 600, San Francisco, California 94105, USA; 3 Program in Medical Ethics, University of California, San Francisco, 521 Parnassus Avenue, Suite C126, Box 0903, San Francisco, California 94143-0903, USA

## Abstract

**Background:**

Shared decision-making has been advocated; however there are relatively few studies on physician preferences for, and experiences of, different styles of clinical decision-making as most research has focused on patient preferences and experiences. The objectives of this study were to determine 1) physician preferences for different styles of clinical decision-making; 2) styles of clinical decision-making physicians perceive themselves as practicing; and 3) the congruence between preferred and perceived style. In addition we sought to determine physician perceptions of the availability of time in visits, and their role in encouraging patients to look for health information.

**Methods:**

Cross-sectional survey of a nationally representative sample of U.S. physicians.

**Results:**

1,050 (53% response rate) physicians responded to the survey. Of these, 780 (75%) preferred to share decision-making with their patients, 142 (14%) preferred paternalism, and 118 (11%) preferred consumerism. 87% of physicians perceived themselves as practicing their preferred style. Physicians who preferred their patients to play an active role in decision-making were more likely to report encouraging patients to look for information, and to report having enough time in visits.

**Conclusion:**

Physicians tend to perceive themselves as practicing their preferred role in clinical decision-making. The direction of the association cannot be inferred from these data; however, we suggest that interventions aimed at promoting shared decision-making need to target physicians as well as patients.

## Background

Shared decision-making between clinicians and patients has been advocated. Some argue that patients have a right to be involved in decisions concerning their health and well-being [1-3], while others use a more utilitarian approach, stating that increased involvement by patients in their health care can lead to improved adherence to management plans and improved health outcomes [4-9].

Whatever the rationale underlying shared decision-making, there is agreement that it can only be achieved when both parties (doctor and patient), commit to sharing the decision-making process [10-12]. In pursuit of this goal, there have been efforts to determine the competencies needed for shared decision-making in clinical practice [13,14], along with attempts to teach and assess these competencies in medical students, doctors in training, and senior clinicians [15-19].

There have also been numerous studies determining patient preferences for various types of decision-making [20-24], and the degree to which those preferences are met [25]. However, there are surprisingly few studies on clinicians' preferences for, and experiences of, different types of clinical decision-making. Qualitative studies have explored perceived barriers to shared decision-making amongst participants [17,26]. In a small study of 45 first year residents, McKeown et al. found that residents thought that patients should play a greater role than the clinician in the decision making process [27]. Charles et al. undertook a survey of all Ontario breast cancer specialists to determine the extent to which respondents reported practising shared decision-making with their patients, their comfort level with this approach, and perceived barriers and facilitators to implementation [28]. We have been unable to identify a population-based survey of physicians from more than one specialty that reports preferences for, and experiences of, decision-making style in their clinical practice.

Such nationally representative data on physician perceptions are needed to help guide interventions aimed at promoting shared decision-making. Many patients are not experiencing their desired role in clinical decision-making [25,29,30]. There are many possible reasons for this, including structural constraints [31,32], perceived lack of time during consultations [28], and limited or inadequate information for patients preventing them from participating meaningfully in decision-making [33,34]. As physicians tend to have more power than patients in the doctor-patient relationship, it is likely that physician preferences and behaviours impact on patient ability to participate in the decision-making process.

As a preliminary step in exploring physician perceptions of shared decision-making and related issues, we undertook a secondary analysis of data from a national survey of U.S. physicians. We sought to determine physician preferences for different styles of clinical decision-making, physician perceptions of the style of clinical decision-making they experienced, and the degree to which physician preference and physicians' perceived experience were congruent. As physicians have an important role in helping patients become adequately informed about their health and health care [35], we assessed physicians' views of their role in encouraging patients to seek information. Finally, because shared decision-making requires time for discussion and deliberation (Table 1) [10], we described physicians' views about the availability of time in patient visits (consultations). We hypothesised that these outcomes would be independently associated with physician characteristics and the socio-economic status of their patients.

**Table 1:** Charles' et al model of medical decision-making*

	Paternalism	Shared decision-making	Consumerism
Information transfer	1-way: From doctor to patient, minimum necessary for informed consent	2-way: doctor provides all medical information needed for decision-making, patient provides information about health utilities	1-way: From doctor to patient, all medical information needed for decision-making
Deliberation	Physician alone, or with other physicians	Physician and Patient (plus potential others)	Patient (plus potential others)
Decision about implementing treatment	Physician	Physician and Patient	Patient

*taken from Charles et al 1999 [11]

## Methods

### Design

Cross-sectional survey of a large, national probability survey of U.S. physicians, undertaken between November 2000 and February 2001 [36,37].

### Sample

Questionnaires were mailed to physicians who currently spent over twenty hours a week on direct patient care randomly selected from the national list of physicians provided by the Medical Marketing Service, Inc (MMS). The sample was stratified by specialty: primary care, medical specialty or surgical specialty. Primary care included Family Practice, General Practice, Internal Medicine and Pediatrics. Ob-Gyn was classified as a surgical specialty.

### Data collection

The questionnaire consisted of closed-ended questions and took approximately 12 minutes to complete. The primary aim of the questionnaire was to ascertain physicians' views on the effects of health information on the Internet or direct to consumer advertising on health outcomes and health service utilisation [36,37]. Questions asked about physician characteristics (age, gender, ethnic origin) and practice characteristics (proportions of patients on Medicaid (U.S. health insurance for eligible poor and disabled persons), from ethnic minority groups, having household incomes of less than $20,000 per annum, and without health insurance; and whether the practice is located in an urban, suburban, small town, or rural setting. All respondents were asked how often they encouraged their patients to look for information about their own medical conditions or treatments, and how often they had enough time to spend with patients. Physicians were also asked, in general, what role they would like to play, and what role they actually played in their patients' health care decisions. Options offered were: "you keep your patients informed, but in general, make health care decisions for them on the basis of what you think is best" (henceforth referred to as "paternalism"); "you discuss options with your patients and their families and then come to a decision together" (henceforth referred to as "shared decision-making"); and "you tell your patients and their families the options, and the pros and cons of each, and then they decide what to do" (henceforth referred to as "consumerism"). Additional physician and practice characteristics were gleaned from the MMS database including specialty, geographic region (East, South, Midwest, and West), whether hospital or office-based, and whether trained in the U.S. or overseas.

### Analysis

#### Weighting

Data were weighted to match the national population of physicians on the MMS database who spend 20 or more hours per week on direct patient care. Factors used in the weighting were data from the MMS database: specialty, year of graduation from medical school, geographic region (East, South, Midwest, West), whether hospital or office-based, and whether trained in the U.S. or overseas.

#### Analytical procedures

The analytic approach focused on evaluating univariate and multivariate relationships with the outcomes of interest: 1) respondents' preferred style of decision-making (preferred style); 2) style of decision-making respondents' reported practicing (perceived style); 3) agreement between preferred and perceived style of decision-making; 4) respondents' perceptions of how often they encouraged patients to look for information; and 5) their perceptions of how often they spent enough time with patients.

We hypothesized that patient socio-economic status and physician characteristics would be independently associated with the outcomes of interest. As we had no direct measures of patient socio-economic status we used respondent's descriptions of their practice characteristics in terms of proportions of patients on Medicaid, from minority groups, having household incomes of less than $20,000 per annum, and without health insurance as proxy measures. We evaluated both groups of variables (physician characteristics and practice characteristics) for their statistical association with the outcome variables.

Univariate relationships between these outcomes and demographic characteristics and health care experiences were assessed using the chi-squared statistic or the Fisher exact test. If a univariate relationship with style of decision-making was found to be significant (p < .05), post-hoc pairwise comparisons (shared decision-making vs. paternalism, shared decision-making vs. informed consumer, and paternalism vs. informed consumer) were run using the Bonferroni procedure to control for Type 1 error.

When results of the univariate analyses suggested that several variables were significantly associated with the outcome of interest, multivariate analyses were performed. An iterative process of forward and backward stepwise regressions was undertaken to determine the most parsimonious model, i.e. the model in which all the independent variables are significantly related to the dependent variable (p < .05), or nearly so (p < .10) while still achieving adequate fit. The Hosmer-Lemeshow Goodness-of-Fit test was applied, and all results of multivariate analyses reported come from final models with adequate fit, defined as p > .2. As all data were weighted, the appropriate procedures to correct p-values and standard errors were undertaken. We used the SVYTAB procedure in STATA to obtain the Rao and Scott F-test p-value, and the SVYLOGIT and SVYMLOGIT procedures in STATA to obtain corrected standard errors for parameter estimates [38].

## Results

### Response rate

Of the 2,000 physicians sent the questionnaire, 38 were ineligible because they were deceased, retired or no longer in practice, and 1,050 returned completed surveys (response rate 53%).

### Characteristics of respondents

Respondents' characteristics are shown in Table 2 in both unweighted and weighted distributions. There is little difference between the unweighted (raw) data, and the weighted data, providing reassurance that the original sample was representative of the national population of U.S. physicians. From this point on, all data presented are weighted.

**Table 2:** Demographic, workload and practice characteristics of respondents

	**Unweighted N (%)**			**Weighted N (%)**		
***Demographic and practice characteristics:***						
***Age***						
< 39	222 (22)			198 (20)		
40 – 49	360 (36)			363 (36)		
50 – 59	248 (25)			248 (25)		
60+	169 (17)			188 (19)		
						
***Gender***						
Female	228 (22)			223 (22)		
Male	808 (78)			812 (78)		
						
***1999 Income from practice***						
$100,000 or less	177 (19)			179 (19)		
$100,001 – $150,000	298 (31)			297 (31)		
$151,001 – $200,000	194 (20)			195 (20)		
$200,001 – $250,000	128 (13)			126 (13)		
$250.001+	162 (17)			160 (17)		
						
***Geographic setting***						
Urban	342 (34)			346 (34)		
Suburban	334 (33)			333 (33)		
Small Town	275 (27)			273 (27)		
Rural	67 (7)			66 (7)		
						
***Geographic Region***						
East	288 (27)			298 (28)		
South	316 (30)			310 (30)		
Midwest	231 (22)			230 (22)		
West	215 (21)			213 (20)		
						
***Type of medical specialty***						
Primary care	404 (39)			406 (39)		
Medical specialty	350 (33)			355 (34)		
Surgical specialty	296 (28)			289 (28)		
						
***Office-based or Hospital-based***						
Office-based	942 (90)			937 (89)		
Hospital-based	108 (10)			113 (11)		
						
***Country of training***						
U.S.	946 (90)			937 (89)		
Foreign	104 (10)			113 (11)		
						

**Respondents' best estimate of the percentage of their patients who were:**	**Unweighted percentiles**			**Weighted percentiles**		
	
	**25**^th^	**50**^th^	**75**^th^	**25**^th^	**50**^th^	**75th**

Uninsured	3	5	13	3	5	13
						
On Medicaid	5	10	25	5	10	25
						
From a minority group	10	20	40	10	20	40
						
Had an annual household income of $20,000 or less	10	15	30	9	15	30
						
**Respondents' best estimate of :**						
Number of hours spent per week in face-to-face contact with patients	24	32	40	24	32	40
						
Number of patients seen per week	50	80	105	50	80	104

### Preferred style of medical decision-making

Of the 1,043 respondents to this question, 75% stated they preferred to share the decision-making with their patients, 14% preferred to make the decisions themselves on the basis of what they thought best for the patient (paternalism) and 11% preferred patients (or their families) to make the decisions (consumerism).

The strength of association between preferred and perceived style was statistically overwhelming (Table 3). This association dominated both the univariate and multivariate analyses, so in order to determine whether any other factors were contributing to the variance, we performed a second logistic regression excluding the style of clinical decision-making respondents perceived themselves as practicing (Table 4).

**Table 3:** Relationship between preferred and perceived style of decision-making for physicians

**Preferred style of decision-making**	**N**	**Perceived style of decision-making**		
		Paternalism N (%)	Shared decision-making N (%)	Consumerism N (%)

Paternalism	142	108 (76)	30 (21)	4 (3)
Shared decision-making	780	39 (5)	710 (91)	31 (4)
Informed Consumer	118	7 (6)	25 (21)	86 (73)

Overall, 87% of physicians perceived themselves as practicing their preferred style of decision-making.

**Table 4:** Conditional Odds Ratios for styles of decision-making preferred by physicians (excluding perceived role)

**Physician characteristics**	Paternalism vs. Shared decision-making*	Consumerism vs. Shared decision-making.**
	COR (95% CI)	COR (95% CI)

***Age***		
28 – 49	1.00	1.00
50+	2.09 (1.41 – 3.11)	1.11 (0.72 – 1.71)
		
***Country of training***		
U.S.	1.00	1.00
Overseas	2.37 (1.34 – 4.19)	0.98 (0.45 – 2.16)
		
***Respondent's type of medical specialty***^*1*^		
Primary Care	1.00	1.00
Medical Specialty	1.32 (0.84 – 2.07)	1.66 (0.97 – 2.87)
Surgical Specialty	0.74 (0.43 – 1.29)	2.56 (1.51 – 4.34)
		
***Percentage of patients from minority backgrounds***		
40% or less	1.00	1.00
> 40%	1.53 (0.96 – 2.45)	1.10 (0.64 – 1.89)
		
***Physician perceptions of frequency of encouraging patients to look for information***		
Often/Sometimes	1.00	1.00
Hardly ever/Never	2.05 (1.33 – 3.17)	0.96 (0.57 – 1.61)
		
***Physician perceptions of frequency of having enough time with patients in visits***		
Often/Sometimes	1.00	1.00
Hardly ever/Never	1.81 (1.03 – 3.18)	1.54 (0.84 – 2.85)

* A Conditional Odds Ratio of > 1 means that physicians in this category had a greater likelihood of preferring paternalism compared to shared decision-making than the referent group.

** A Conditional Odds Ratio of > 1 means that physicians in this category had a greater likelihood of preferring consumerism compared to shared decision-making than the referent group.

^1^Physicians in surgical specialties were less likely than physicians in medical specialties to prefer paternalism compared to shared decision-making (OR 0.56; 95% CI 0.33 – 0.97), but no more likely to prefer consumerism compared to shared decision-making (OR 1.54; 95% CI 0.94 – 2.51).

In all sub-groups of physicians, over 70% of respondents reported preferring to share clinical decision-making with their patients. Physician characteristics were independently associated with respondent's preferences for particular styles of clinical decision-making. Older doctors (aged 50 or older) and doctors trained overseas were more likely to prefer paternalism than shared decision-making. Physicians who seldom encouraged patients to look for information and who felt they did not have enough time with patients in visits were also more likely to prefer paternalism to shared decision-making. Respondents from surgical specialties were more likely to prefer consumerism to shared decision-making than physicians working in either primary care or a medical specialty.

### Perceived style of medical decision-making

Of the 1,040 respondents to this question, 73% stated that they shared the decision-making with their patient, 15% said they made the decision on behalf of the patient, and 12% left the decision-making to the patient or the patient's family. Once again, the initial multivariate analysis of factors associated with physicians' perceptions of decision-making in a clinical encounter was dominated by physician preferences. We therefore performed a second multivariate analysis excluding physicians' preferences in order to explore whether other factors also played a role (Table 5).

**Table 5:** Conditional Odds Ratios for perceived style of decision-making (excluding preferred style)

	Paternalism vs. Shared decision-making*	Consumerism vs. Shared decision-making**
	COR (95% CI)	COR (95% CI)

***Age***		
28 – 49	1.00	1.00
50+	1.69 (1.15 – 2.47)	0.96 (0.62 – 1.49)
		
***Country of training***		
U.S.	1.00	1.00
Overseas	2.20 (1.24 – 3.90)	2.07 (1.08 – 3.99)
		
***Geographic setting of practice***^*1*^		
Urban	1.00	1.00
Suburban	1.37 (0.84 – 2.25)	0.48 (0.28 – 0.81)
Small town	1.15 (0.68 – 1.95)	0.39 (0.22 – 0.72)
Rural	0.47 (0.16 – 1.44)	0.71 (0.29 – 1.75)
		
***Respondent's type of medical specialty***^*2*^		
Primary Care	1.00	1.00
Medical Specialty	1.20 (0.78 – 1.84)	1.16 (0.64 – 2.09)
Surgical Specialty	0.56 (0.32 – 0.96)	2.66 (1.58 – 4.47)
		
***Percentage of patients from minority backgrounds***		
40% or less	1.00	1.00
> 40%	2.22 (1.40 – 3.51)	0.79 (0.45 – 1.42)
		
***Physician perceptions of frequency of encouraging patients to look for information***		
Often/Sometimes	1.00	1.00
Hardly ever/Never	1.75 (1.15 – 2.68)	1.19 (0.71 – 2.00)
		
***Physician perceptions of frequency of having enough time with patients in visits***		
Often/Sometimes	1.00	1.00
Hardly ever/Never	1.75 (1.02 – 2.99)	0.97 (0.47 – 2.02)

* A Conditional Odds Ratio of > 1 means that physicians in this category had a greater likelihood of perceiving themselves as practicing paternalism compared to shared decision-making than the referent group.

** A Conditional Odds Ratio of > 1 means that physicians in this category had a greater likelihood of perceiving themselves as practicing consumerism compared to shared decision-making than the referent group.

^1^There were no other significant findings for geographic setting, whichever setting was used as the referent point.

^2^Physicians in surgical specialties were less likely to perceive themselves as practicing paternalism than physicians in medical specialties (OR 0.46; 95% CI 0.27 – 0.80), and more likely to perceive consumerism (OR 2.29; 95% CI 1.36 – 3.88).

This analysis revealed that both physician and practice characteristics were associated with their perceptions of clinical decision-making. Older physicians (50 or older) were more likely to perceive themselves as practicing paternalism. Physicians trained overseas were less likely to perceive themselves as practicing shared decision-making than U.S. trained physicians and more likely to perceive either paternalism or consumerism. Respondents from surgical specialties were less likely to perceive paternalism and more likely to perceive consumerism than physicians from either medical or primary care specialties. Physicians who seldom encouraged patients to look for information and who felt they seldom had enough time with patients during visits were more likely to perceive paternalism.

Practice characteristics were also associated, with physicians with high proportions of minority patients more likely to perceive paternalism and physicians working in urban areas more likely to perceive consumerism.

### Congruence between preferred and perceived style of medical decision-making

87% of doctors perceived themselves as practicing the style of decision-making they preferred (Table 3). On multi-variate analysis, age, specialty and proportion of patients from minority backgrounds were the only factors independently associated with perceiving themselves as practicing the preferred style. Younger physicians, physicians in primary care, and physicians with less than 40% of patients from minority backgrounds were all more likely to perceive themselves as practicing their preferred style (Table 6).

**Table 6:** Odds ratios for factors associated with physicians perceiving themselves as practicing their preferred role

	Odds Ratio	95% Confidence Interval
***Age***		
28 – 49	1.00	
50+	0.57	0.37 – 0.88
		
***Respondent's type of medical specialty***		
Primary Care	1.00	
Medical Specialty	0.55	0.32 – 0.93
Surgical Specialty	0.50	0.29 – 0.87
		
***Percentage of patients from minority backgrounds***		
40% or less	1.00	
> 40%	0.56	0.32 – 0.97

### Physician's role in encouraging patients to seek information

Physicians were asked how often they encouraged their patients to look for information. Physicians felt they did this fairly frequently, with 32% stating they did this "often", 45% stating they did this "sometimes", 19% saying they "hardly ever" did, and only 4% saying they "never" encouraged patients to look for information.

Physicians who preferred either a shared decision-making or consumerist style of decision-making were more likely to encourage patients to look for information, as were physicians who felt they did not have enough time with patients (Table 7).

**Table 7:** Odds ratios for physicians encouraging patients to look for information often or sometimes

	Odds Ratio	95% CI
Preferred role in clinical decision-making*		
Paternalism	1.00	
Shared decision-making	2.78	1.68 – 4.60
Consumerism	3.81	1.63 – 8.90
		
***Physician perceptions of frequency of having enough time with patients during a visit***		
Often/Sometimes	1.00	
Hardly ever/Never	2.00	1.07 – 3.76

* Physicians who preferred consumerism were no more likely to encourage patients to look for information than physicians who preferred shared decision-making (OR 1.37; 95% CI 0.65 – 2.88).

### Provision of adequate time in visits

48% of physicians felt they often spent enough time with patients, 40% stated they sometimes did, 10% of physicians felt they hardly ever and 1% stated they never spent enough time with patients (Table 8).

**Table 8:** Odds ratios for physicians perceiving that they often or sometimes have enough time to spend with patients in visits

	Odds Ratios	95% Confidence Intervals
***Type of specialty***^*1*^		
Primary care	1.00	
Medical specialty	0.85	0.54 – 1.34
Surgical specialty	2.11	1.12 – 3.96
		
***% Patients with annual incomes < $20,000***		
30% or less	1.00	
Over 30%	0.49	0.32 – 0.76
		
***Preferred role in clinical decision-making***^*2*^		
Paternalism	1.00	
Shared decision-making	1.83	1.04 – 3.25
Consumerism	1.03	0.48 – 2.22
		
***Physician perceptions of frequency of encouraging patients to look for information***.		
Often/Sometimes	1.00	
Hardly ever/Never	2.73	1.49 – 4.98

^1^Physicians in surgical specialties were also more likely to perceive themselves as often or sometimes having enough time when compared with physicians in medical specialties (OR 2.5; 95% CI 1.3 – 4.6)

^2^Physicians who preferred shared decision-making were non-significantly more likely to perceive they often or sometimes spent enough time with patients in visits compared to physicians who preferred consumerism (OR 1.78; 95% CI 0.96 – 3.28).

Both physician and practice characteristics were associated with respondents' perceptions of adequacy of time spent with patients in visits. Surgeons were more likely than medical or primary care specialists to feel they often or sometimes spent enough time with patients. Physicians with high proportions of low-income patients were less likely to feel they had enough time than those with fewer low-income patients. Preferring shared decision-making was associated with feeling there was enough time in visits. Physicians who seldom encouraged patients to look for information were also more likely to feel they had enough time in visits.

## Discussion

### Main findings

These are the first data from a large nationally representative sample of physicians to explore physician perceptions of their preferred and perceived role in clinical decision-making. The main findings are that three-quarters of physicians prefer sharing decision-making with their patients, with the remaining one-quarter almost equally divided between the paternalist and consumerist approaches. Their preference is by far the strongest predictor of the role they perceive themselves as playing in clinical decision-making, with 87% of respondents perceiving themselves as practicing their preferred role. Physicians expressing a preference for shared decision-making were more likely to perceive themselves as practicing their preferred style (91%) than doctors who preferred paternalism or consumerism (76% and 73%, respectively).

As expected, preferring shared decision-making is also associated with encouraging patients to look for information, and feeling that there is usually enough time during visits. Paternalism is associated with the absence of these conditions and is more prevalent among older and non-US trained physicians. Consumerism is more prevalent among surgeons and is associated with encouraging patients to look for information on their own, but has no statistical association with perception of sufficient consultation time.

### Relationship with existing literature

Our results are consistent with those of Charles et al, who undertook a cross-sectional survey of Ontario breast cancer specialists in 1998. In this sample of 322 surgeons and 130 oncologists, over four-fifths reported high levels of comfort with a shared decision-making approach. These physicians had been exposed to many messages about the importance of patient participation in treatment decision-making, and the authors suggest that this may have affected the generalisability of their results. Between one-half and two-thirds of respondents reported actually using a shared decision-making approach, citing difficulties with time and lack of patient information as barriers to patient involvement in treatment decision-making [28].

### Methodological issues

The main strength of this work is the nature of the sample: a nationally representative probability sample of clinicians practicing in the U.S. Despite the moderate response rate (53%), the sample can be taken to be representative of the total population of U.S. physicians on two grounds. Firstly, differences between the weighted and unweighted sample are small, suggesting that the unweighted sample closely reflected the total population. Implementation of weighting ensured that any substantial bias due to non-response was adjusted for. Secondly, systematic non-response bias is unlikely, as the main purpose of the survey (and majority of questions included) related to respondent experience of patients bringing in information from the Internet or direct-to-consumer-advertising. As these data are generated by a national sample, they are likely to generalise to the total population of physicians in the U.S, currently engaged in direct patient care for more than 20 hours a week. They are not limited to a specific institution, speciality, or state.

The definitions of paternalism, shared decision-making, and consumerism used in the questionnaire are consistent with the definitions conceptualised by Charles et al [10,11]. However, social desirability may have influenced responses to the questionnaire, despite the assurances of anonymity. As all these data are entirely self-reported, we have no objective or external measure of the findings reported here. It has been shown that patients and doctors may have discordant reports of the style of decision-making in any given consultation [39]. Another limitation of our data is that we asked about physician preferences and experiences in general, not at the level of individual visits (consultations). It is not likely that any of the physicians sampled used only one decision-making style in all consultations. We were not able to explore the effect of individual patients in specific visits, and are likely to have underestimated the influence of the patient in affecting physician behaviours.

Finally, a cross-sectional survey such as this one, can only demonstrate associations. It cannot indicate causality. Alternative interpretations of these data are possible, such as physicians (like patients) preferring what they are used to [40], or patients demanding shared decision-making from their physicians, who then become accustomed to this style, and hence report preferring it. The inability of cross-sectional data to indicate the direction of a relationship is also pertinent to interpreting the relationship between perceptions of adequacy of time in consultations and physician role in encouraging patients to seek information outside the consultation with preferred and perceived role in clinical decision-making.

## Conclusion

Shared decision-making requires a commitment from both parties (patient and doctor). These data confirm the importance of engaging both the physician and the patient in initiatives to promote shared decision-making. Interventions to enhance shared decision-making should address physician concerns about adequate time during consultations and try to assure that patients have adequate access to health information outside the consultation.

## Competing interests

The author(s) declare that they have no competing interests.

## Authors' contributions

EM conceived the paper, wrote the first and final drafts. LP developed the analytical strategy, and supervised the analysis. MW undertook the analysis. BL had responsibility for the original study which generated the data, and contributed to the conceptualisation of this paper. All authors contributed to the drafting process, all authors have seen and agreed the final version.

## Pre-publication history

The pre-publication history for this paper can be accessed here:


